# Dendritic cell phenotype in severe asthma reflects clinical responsiveness to glucocorticoids

**DOI:** 10.1111/cea.13061

**Published:** 2017-12-18

**Authors:** E. S. Chambers, A. M. Nanzer, P. E. Pfeffer, D. F. Richards, A. R. Martineau, C. J. Griffiths, C. J. Corrigan, C. M. Hawrylowicz

**Affiliations:** ^1^ MRC and Asthma‐UK Centre for Allergic Mechanisms in Asthma King's College London London UK; ^2^ Asthma UK Centre for Applied Research Centre for Primary Care and Public Health Blizard Institute Queen Mary, University of London London UK; ^3^ Present address: Division of Infection and Immunity University College London London UK; ^4^ Present address: William Harvey Research Institute Queen Mary University of London London UK

**Keywords:** asthma, dendritic cell, glucocorticoid, steroid resistant, steroid sensitive, T cells

## Abstract

**Background:**

Subsets of patients with severe asthma remain symptomatic despite prolonged, high‐dose glucocorticoid therapy. We hypothesized that the clinical glucocorticoid sensitivity of these asthmatics is reflected in differences in peripheral blood dendritic cell subsets.

**Objective:**

To compare peripheral blood leucocyte populations using flow cytometry at baseline and after 2 weeks of systemic glucocorticoid (steroid) treatment to identify immunological differences between steroid‐sensitive (SS) and steroid‐resistant (SR) asthmatics.

**Methods:**

Adult severe asthmatics (SS n = 12; SR n = 23) were assessed for their response to 2 weeks of therapy with oral prednisolone. Peripheral blood was obtained before and after therapy and stained for lymphocyte (CD3, CD19, CD4, CD8 and Foxp3) and dendritic cell markers (Lineage negative [CD3, CD14, CD16, CD19, CD20, CD56], HLA‐DR+, CD304, CD11c, ILT3 and CD86).

**Results:**

A higher median frequency of myeloid DCs (mDCs) but not plasmacytoid DCs (pDCs) was observed in the blood of SR as compared to SS asthmatics (*P* = .03). Glucocorticoid therapy significantly increased median B cell, but not T cell numbers in both cohorts, with a trend for increased numbers of Foxp3+ Tregs in SS (*P* = .07), but not SR subjects. Oral prednisolone therapy significantly reduced the median numbers and frequencies of total DCs and pDCs in both SS and SR asthmatics. Interestingly, the expression of HLA‐DR and ILT3 was also reduced on pDCs in all patients. In contrast, therapy increased the median frequency of mDCs in SS, but reduced it in SR asthmatics.

**Conclusions:**

Myeloid DC frequency is elevated in SR compared with SS asthmatics, and mDC shows a differential response to oral prednisolone therapy.

## 1 INTRODUCTION

Asthma is characterized by airway hyperresponsiveness, T cell‐mediated inflammation, mucus hypersecretion and airway remodelling. Glucocorticoids (steroids) represent the cornerstone of contemporary asthma therapy, improving lung function and asthma symptoms in many patients (steroid sensitive, SS). Beyond these global features of asthma pathophysiology, however, evidence continues to accumulate about the heterogeneity of the disease in terms of its natural history, the nature and degree of associated airways inflammation and its responsiveness to steroid therapy.^1^ Indeed, it has been evident for some time that there exists a subgroup of asthmatic patients showing no demonstrable clinical benefit, at least in terms of short‐ to medium‐term improvement in lung function, in response to physiologically appropriate systemic steroid therapy (steroid resistant, SR). These patients are at risk of high morbidity and mortality, and as such pose an unmet clinical need.

Several mechanisms have been proposed to contribute to steroid non‐responsiveness at the cellular level, particularly in T cells, including overexpression of pro‐inflammatory transcriptional regulators such as NFκB and AP‐1, increased expression of histone deacetylases, polymorphisms of the IL‐10 gene, elevated expression of the dominant negative isoform of the glucocorticoid receptor GRβ, overexpression of Th2 cytokines and vitamin D insufficiency.^2^, ^3^, ^4^, ^5^, ^6^, ^7^, ^8^, ^9^, ^10^ More recent evidence also suggests that IL‐17A overexpression can be both a marker and a risk factor for severe and SR asthma.^11^, ^12^, ^13^, ^14^, ^15^, ^16^, ^17^

Notwithstanding these observations, there have been few if any studies of the dynamic effects of systemic glucocorticoid therapy in vivo, and in particular, whether glucocorticoids exert differential effects on lymphocyte frequency and functional differentiation in SS as compared with SR asthmatics, which may contribute to their different clinical responsiveness. Elucidating the mechanisms behind steroid‐resistant disease is vital to improving treatments for these patients. As asthmatic inflammation is postulated to be fundamentally lymphocyte driven, the cell types of particular interest, which may play a role in differential steroid responsiveness in SS and SR asthmatics, include lymphocytes such as CD4+ T cells including regulatory T cells (Tregs), CD8+ T cells, B cells and dendritic cells (DCs).^8^, ^18^ Dendritic cells are sentinels of the immune system, as they are early sensors of “stress” or pathogens, and when such signals are detected, they will mature and migrate to local lymph nodes to initiate an adaptive immune response. DCs present antigen‐derived peptides (such as those derived from processed allergens) bound to MHC molecules as well as costimulatory molecules (CD80 and CD86) to T cells which bind to the T cell receptor (TCR) and CD28, respectively, resulting in T cell activation and functional differentiation. In contrast inhibitory receptors, such as immunoglobulin‐like transcript 3 (ILT3), are associated with a more tolerogenic DC phenotype, as an expression of ILT3 has been shown to promote the generation of Foxp3+ Treg.^19^, ^20^, ^21^

There are 2 major subsets of DCs: myeloid dendritic cells (mDCs) and plasmacytoid dendritic cells (pDCs). The study of DCs is relevant to asthma as mDCs are resident in the airway mucosa and survey the airway lumen for antigen and particulates.^22^ Myeloid DCs have been shown to induce a Th2‐inflammatory response in the airways of asthmatics in response to aeroallergen exposure, which results in eosinophilic infiltration and disease exacerbation.^23^ Plasmacytoid DCs express high levels of IL‐3R, CD123, and MHC class II, and very low levels of costimulatory molecules, pDCs are specialized for production of high levels of anti‐viral type 1 interferon production and prime Th1 effector generation.^24^ However, pDCs may also play a role in generating tolerance to inhaled antigens,^25^ to protect the epithelium from damage caused by aberrant T cell responses to otherwise harmless antigens and particulates.

The aim of this study was to investigate differences in peripheral blood DC frequency, number and phenotype in clinically defined SS and SR asthma patients, and the impact of 2 weeks of oral prednisolone treatment on these parameters. We further investigated whether this was associated with any changes in lymphocyte frequency and numbers. We hypothesized that SS patients demonstrate a less activated, more tolerogenic DC phenotype in comparison with SR patients reflected in the frequency of their circulating, regulatory T cells, and that glucocorticoid treatment enhances this phenotype in SS individuals.

## 2 MATERIALS AND METHODS

### 2.1 Subjects

All patients recruited provided written, informed consent (National Research Ethics Committee 08/H0804/84). Eligible patients had a specialist physician diagnosis of moderate to severe asthma for at least 6 months and were managed with inhaled steroids and short‐ and long‐acting bronchodilators corresponding to therapy step 3 or 4 of the 2012 British Thoracic Society (BTS) guidelines on the management of asthma.^26^ At recruitment, all patients were required to demonstrate airways obstruction with a pre‐bronchodilator FEV_1_ of <80% of the predicted value, and clear reversibility of this obstruction in response to short‐acting bronchodilator (>12% improvement following inhalation of 400 μg of salbuterol). In addition, all patients had undergone a detailed assessment to exclude a diagnosis other than asthma and comorbidities affecting asthma control. Patients had not been exposed to systemic steroids for at least 4 weeks prior to the study. Patients on immunotherapy, smokers or patients who had symptoms and signs suggesting a respiratory tract infection or any asthma exacerbation during the 4 weeks prior to enrolment for the study were also excluded.

Eligible patients were characterized as steroid sensitive or resistant as defined by a ≥10% or <10% improvement, respectively, in baseline FEV_1_ following a pharmacodynamically standardized, a 14‐day course of oral prednisolone (40 mg/1.73 m^2^/d; Wockhardt UK Ltd, Wrexham, UK).^27^ Ongoing inhaled therapy was not altered during this treatment. Routine spirometry was measured before and after the course of prednisolone using a PC based spirometer and software (WinspiroPRO Medical International Research, Rome, Italy). Peripheral venous blood was obtained from all study subjects and collected into tubes containing sodium citrate and was directly stained as described below.

### 2.2 Flow cytometry

The following antibodies were used for ex vivo phenotyping of peripheral blood obtained from asthma donors: CD3, CD4, CD8, CD11c, CD14, CD16, CD19, CD20, CD56, CD86 and HLA‐DR (SK7, RPA‐T4, RPA‐T8, B‐ly6, MφP9, 3G8, HIB19, 2H7, NCAM16.2, 2331[FUN‐1] and L243, respectively; BD Biosciences, Oxford, UK), ILT3 and Foxp3 (ZM4.1 and PCH101, respectively; eBiosciences, Hatfield, UK) and CD304 (BDCA‐4) (AD5‐17F6; Miltenyi Biotec Bisley, UK). Red blood cells were lysed following staining with BD FACS lysing solution. Foxp3 staining was performed as previously described.^18^ Samples were subsequently processed on a FACSCalibur flow cytometer running CellQuest Pro software (BD Biosciences), which was also used for analysis. A live dead stain was not used as the flow cytometry staining was performed on whole blood ex vivo.

Absolute and differential blood leucocyte counts were performed routinely using a LH750 haematology analyser (Beckman Coulter, CA, USA) and analysed in conjunction with flow cytometric data to calculate cell numbers.

### 2.3 Cytometric bead array

Serum samples collected from the asthmatics were analysed for the following cytokines (IL‐1β, IL‐5, IL‐8, IL‐6, IL‐13, IL‐17A, IFNγ, GM‐CSF and TNFα) using a cytometric bead array according to the manufacturer's instructions (BD Bioscience). The lower limit of detection was 1.5 pg/mL.

### 2.4 Statistics

Data were assessed for equivalence to a Gaussian distribution and equality variance after which the appropriate parametric or nonparametric test was performed (see individual figure legends). Differences were considered significant at the 95% confidence level. Box‐and‐whisker plots represent the median, interquartile range and 10th to 90th percentiles, with outliers outside the 10th to 90th percentiles shown as individual points.

## 3 RESULTS

### 3.1 Study patients and clinical steroid responsiveness

Clinical and demographic details of the study patients have been published previously.^28^ Briefly, the asthmatic patients differentiated as steroid sensitive (SS) or SR based on their FEV_1_ response to a pharmacodynamically standardized course of oral prednisolone were otherwise well matched in terms of age, ethnicity, sex, atopic status, body mass index, total inhaled steroid dosage and baseline FEV_1_. Mean (95% CI) FEV_1_ significantly improved from 56.0 (47.4%‐64.6%) to 70.8 (62.6%‐79.0%) of the predicted value following prednisolone in the SS asthmatics (*P* < .0001), whereas in the SR patients there was no significant change: 61.3 (55.3%‐67.3%) to 59.7 (52.8%‐66.5%) (*P* = .18) (Table S1). Demographic data on healthy control subjects studied are also provided in Table S1.

### 3.2 Oral prednisolone significantly increases the number of peripheral blood B cells

Cell surface staining was performed on peripheral blood cells of severe asthmatics and healthy control subjects. No differences in the frequency of lymphocyte populations between healthy controls and the severe asthmatics were observed at baseline (Table 1).

**Table 1:** The frequency of lymphocytes is similar between severe asthmatics and healthy controls

	Healthy	Severe asthma	*P* value
CD19+ lymphocytes	7.32 (5.7‐8.9)	8.45 (7.2‐9.8)	.37
CD3+ lymphocytes	59.70 (50.1‐69.3)	58.99 (55.1‐62.9)	.87
CD4+ T cells	62.15 (55.3‐67.0)	65.10 (61.1‐69.06)	.47
CD8+ T cells	29.91 (23.2‐36.7)	30.04 (26.2‐33.9)	.97
CD3+/CD19+ ratio	8.96 (5.9‐12.0)	8.41 (7.1‐9.7)	.70
Foxp3+ CD4+ T cells	7.99 (7.2‐8.8)	7.88 (6.9‐8.8)	.46
	n = 10	n = 35	

Healthy controls and severe asthmatics were assessed at baseline for lymphocyte populations including B Cells (CD19+), T cells (CD3+) and for T regulatory cells (Foxp3+CD4+ T cells). Data are shown as mean ± 5%‐95% confidence intervals.

**Figure 1:**
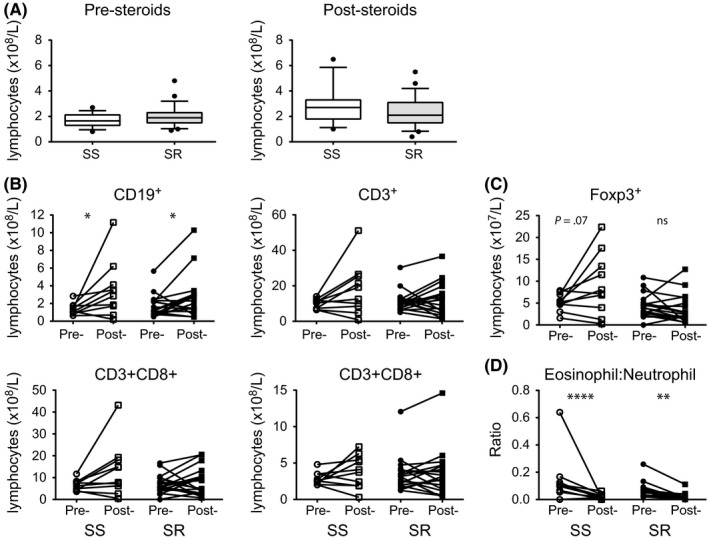
Absolute lymphocyte counts in the peripheral blood of steroid‐sensitive (SS) and steroid‐resistant (SR) severe asthmatics before (pre‐) and after (post‐) a 2‐week course of oral prednisolone. A, total lymphocytes; B, total B cells (CD19+), T cells (CD3+), CD4+ and CD8+ T cells; C, total Foxp3+ CD4+ T cells; D, eosinophil to the neutrophil ratio in the peripheral blood of SS and SR severe asthmatics. Data assessed by Wilcoxon matched‐pairs test *P < .05, **P < .01, ****P < .001

Cell surface staining was also performed on SS and SR asthmatics before and after the 2‐week course of prednisolone, which was used to define their responder status. As previously reported,^28^ there were no significant differences between the groups in the median numbers of circulating total B cells, T cells, CD4+ T cells or CD8+ T cells prior to oral prednisolone treatment. Following treatment with oral prednisolone, no significant change in the median total numbers of circulating lymphocytes in the SR and SS asthmatic was seen (Figure 1A). The median total numbers of circulating B cells, as defined by CD19+ cell surface expression, significantly increased in both groups post‐prednisolone without significant change in the median total numbers of circulating T cells (as defined by CD3+ cell surface expression), CD4+ T cells or CD8+ T cells (Figure 1B). Prednisolone therapy was also associated with a trend towards an increase in the median total number of circulating Foxp3+ T regulatory cells in the SS, but not the SR asthmatics (Figure 1C). This staining was performed ex vivo on whole blood to ascertain that we were looking at “real” Treg populations, as it has been reported that Foxp3 can be induced in T effector cells by activation. Therefore, in a number of severe asthmatics, we included in the staining protocol antibodies specific for CD25 and CD127, as cells that are CD25high and CD127low are reported to represent Treg cells with suppressive function.^29^ The Foxp3+ CD4+ T cells that we identified as Tregs had >90% of cells falling in the gate CD25^hi^CD127^lo^, whereas the Foxp3‐ T cells had only 2.9% of cells falling in the gate (Figure S1A). Also, when the frequency of CD4+ Foxp3+ cells was correlated with the frequency of CD127^lo^CD25^hi^ T cells, there was a significant positive correlation between these 2 populations (*r* = .752 *P* < .0001) strongly suggesting that we are looking at “true” Treg cells (Figure S1B). The MFI of Foxp3 in the Foxp3+ Treg cells is also shown, although no difference was observed between the 2 groups (Figure S1C).

No difference in the eosinophil and neutrophil numbers was previously reported between SS and SR asthmatics pre‐steroids.^28^ Here we show that there is a significant reduction in the ratio of eosinophils to neutrophils in both SS and SR asthmatics after prednisolone treatment (Figure 1D).

### 3.3 Increased frequency of myeloid dendritic cells in the peripheral blood of steroid‐resistant as compared to steroid‐sensitive asthmatics

**Figure 2:**
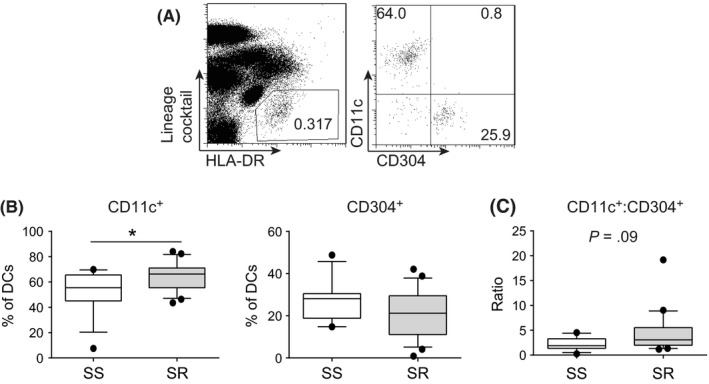
Increased frequency of mDCs in the peripheral blood of steroid‐resistant (SR) as compared to steroid‐sensitive (SS) severe asthmatics. A, example gating of dendritic cell (DC) gating: DCs were identified as being lineage cocktail negative (CD3, CD14, CD16, CD19, CD20 and CD56) and HLA‐DR+ and then subsequently either CD11c+ (mDCs) or CD304+ (pDCs); B, cumulative data of frequency of mDCs and pDCs; and C, the ratio of CD11c+ as compared to BDCA‐4+ DCs in the peripheral blood of SS and SR severe asthmatics prior to any glucocorticoid treatment. Data assessed by Mann‐Whitney U statistical test *P < .05

Dendritic cells (DCs) are sentinels of the immune system; DCs were characterized as being lineage cocktail (CD3, CD14, CD16, CD19, CD20 and CD56) negative and HLA‐DR+ cells. Two major DC subsets, myeloid and plasmacytoid (mDC and pDC, respectively), can be distinguished based on cell surface expression of 2 well‐characterized molecules CD11c for mDCs and CD304 (BDCA‐4; Neuropilin 1) for pDCs^30^ (Figure 2A). The median frequency of CD11c+ mDCs, within the Lineage‐HLA‐DR+ gate, was significantly elevated in the peripheral blood of the SR as compared to the SS asthmatics, whereas there was no significant difference in the median frequencies of CD304 pDCs between the 2 patient groups (Figure 2B). This resulted in a trend (*P* = .09) towards an increased ratio of mDC:pDC cells in the SR as compared to the SS asthmatics (Figure 2C).

**Figure 3:**
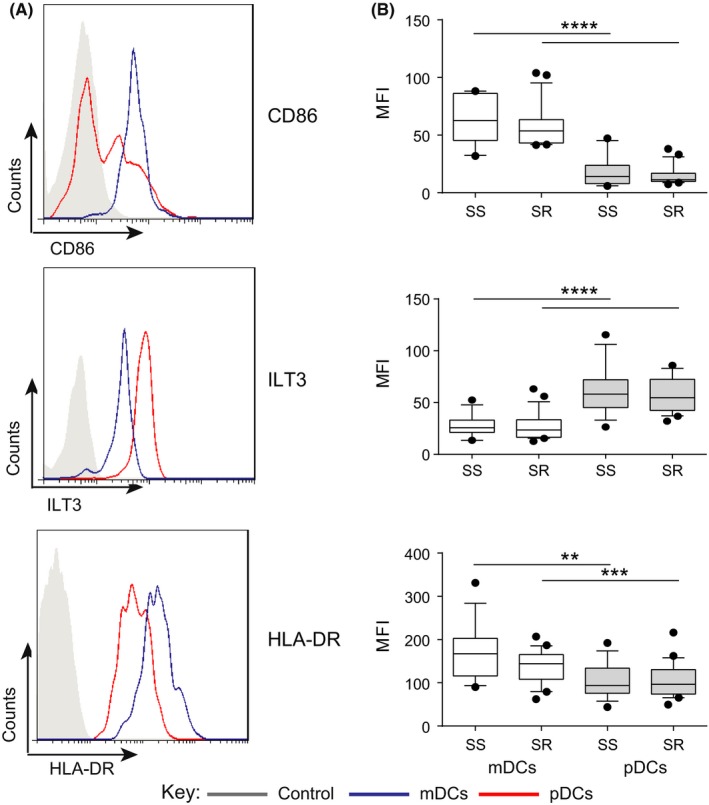
Expression of surface CD86, ILT3 and HLA‐DR on myeloid (mDCs) and plasmacytoid (pDCs) dendritic cells in the peripheral blood of steroid‐sensitive (SS) and steroid‐resistant (SR) asthmatics. Expression of costimulatory and inhibitory receptors on DCs was assessed by flow cytometric analysis in SS and SR patients at baseline prior to glucocorticoid treatment. A, example histograms of CD86 (top), ILT3 (middle) and HLA‐DR (bottom) expression on mDCs (blue) and pDCs (red) and fluorescence minus one (FMO) control (grey shaded); B, cumulative data on mDCs (white) and pDCs (grey) in the peripheral blood of SS and SR severe asthmatics. Data assessed by paired *t* test **P < .01, ***P < .001, ****P < .0001

We also measured cell surface expression of HLA‐DR and the costimulatory molecule CD86 on mDCs and pDCs, as well as that of the Immunoglobulin‐like transcript 3 (ILT3), an inhibitory receptor implicated in promoting tolerance in T cells.^19^, ^20^, ^21^ Myeloid DCs expressed significantly higher surface CD86 and HLA‐DR, as measured by median fluorescence intensity, but lower surface ILT3 as compared to pDCs (Figure 3A; *P* < .001 in each case). We observed no significant difference, however, in the median intensity of expression of these cell surface molecules in the SS vs SR asthmatic patients (Figure 3B).

### 3.4 Prednisolone treatment significantly decreased total dendritic cells and plasmacytoid dendritic cells frequencies

**Figure 4:**
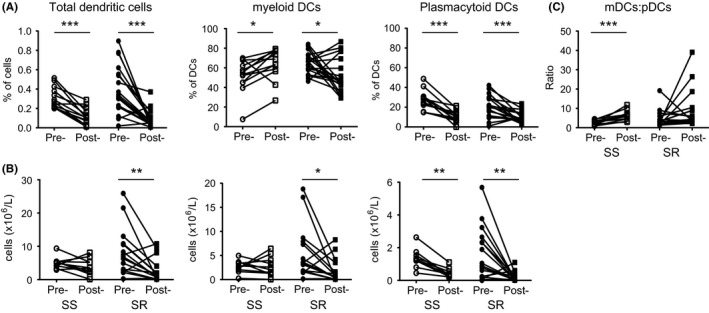
Reduced frequencies and numbers of dendritic cells (DC)s in the peripheral blood of asthmatics following prednisolone therapy. Steroid‐sensitive (SS) and steroid‐resistant (SR) asthmatics were assessed before and after a 2‐week course of oral prednisolone therapy (A) frequencies and (B) total numbers of total DCs, mDCs and pDCs; (C) the ratio of CD11c+ as compared to CD304+ DCs in the peripheral blood of steroid‐sensitive (SS; white) and steroid‐resistant (SR; grey) severe asthmatics before and after 2 weeks of prednisolone therapy. Data assessed by Mann‐Whitney U test *P < .05, **P < .01, ***P < .001

As shown in Figure 4, a 2‐week course of oral prednisolone resulted in a significant reduction in the median frequencies of total DCs in the leucocyte population of both SS and SR asthmatics (Figure 4A). This was reflected in a reduction in the median absolute number of total DCs in SR asthmatics (*P* < .01), but not in SS asthmatics (*P* = .23; Figure 4B). There was a significant reduction in the median absolute number and frequency of pDCs in both SS and SR asthmatics. In contrast, quite different effects of prednisolone were observed on mDCs in the two severe asthma groups. In the SR asthmatics, there was a significant reduction in the median frequency and number of peripheral blood mDCs following prednisolone therapy. Conversely, in the SS asthmatics, there was a significant increase in the median frequency (*P* < .05), which is most likely due to the decreased frequency of pDCs in the Lineage‐HLA‐DR+ gate. This is reflected in the lack of change in the median absolute number (*P* = .77) of mDCs following prednisolone therapy (Figure 4A and B). Interestingly, these changes in DC subtypes resulted in a significant increase (*P* < .001) in the median overall ratio of mDCs to pDCs in the SS, but not the SR patients (Figure 4C).

The expression of surface CD86, HLA‐DR and ILT3 was also measured on peripheral blood mDCs and pDCs from the SS and SR asthmatics before and after prednisolone therapy (Table 2). Steroid therapy was associated with reduced expression of ILT3 and HLA‐DR on pDCs in both the SS and SR asthmatics, whereas there was no significant effect on CD86 expression. There was no significant effect of steroids on the ILT3 expression on mDCs. There was, however, a suggestion of a differential effect of prednisolone on mDC cell surface expression of CD86 and HLA‐DR in the SS, as compared with the SR asthmatic patients: there was a trend towards reduced expression of CD86 (*P* = .088) and HLA‐DR (*P* = .065) on mDCs in the SS patients which was not observed in the SR patients (Table 2).

**Table 2:** Expression of immunoglobulin‐like transcript 3 (ILT3) and costimulatory molecules on dendritic cells (DC)s before and after corticosteroid therapy

	Steroid sensitive		*P* value	Steroid refractory		*P* value
	Pre‐steroids	Post‐steroids		Pre‐steroids	Post‐steroids	
pDCs						
ILT3 expression	63.44 (48.56‐78.32)	43.64 (32.3‐55.0)	**.028**	60.33 (48.8‐71.87)	31.89 (26.4‐37.4)	**<.0001**
CD86 expression	21.59 (9.9‐33.31)	19.55 (3.2‐35.9)	.693	14.13 (10.5‐17.7)	17.74 (11.6‐23.7)	.251
HLA‐DR expression	105.0 (82.3‐127.7)	76.03 (51.2‐100.8)	**.012**	112.2 (89.7‐134.7)	80.28 (62.6‐97.9)	**.032**
mDCs						
ILT3 expression	27.82 (21.0‐34.6)	26.38 (22.0‐30.8)	.700	27.24 (21.4‐33.1)	26.37 (22.0‐30.8)	.781
CD86 expression	63.19 (50.1‐76.3)	51.29 (38.8‐63.8)	.088	57.72 (41.9‐98.7)	52.82 (41.9‐67.4)	.258
HLA‐DR expression	170.1 (130.4‐209.9)	129.2 (98.9‐159.4)	.065	161.6 (107.1‐216.1)	132.2 (111.3‐153.0)	.262

Mean fluorescence intensity (MFI) of cell surface expression of CD86, HLA‐DR and inhibitory receptor ILT3 on pDCs (CD304+; top) and mDCs (CD11c+, bottom) before and after 2 weeks of prednisolone therapy. Data are shown as mean ± 5%‐95% confidence intervals.

### 3.5 Steroid treatment does not significantly alter the serum cytokine profile

**Figure 5:**
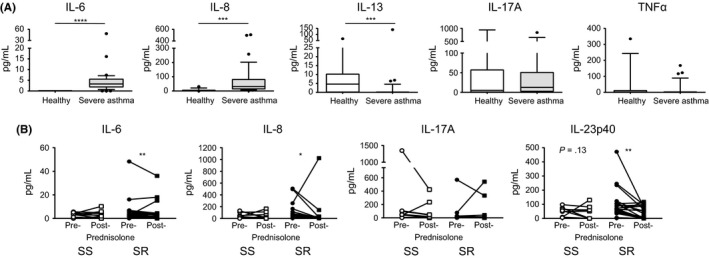
A reduction in serum IL‐6, IL‐8 and IL‐23p40 in SR asthmatics after prednisolone treatment. Serum cytokine concentrations were assessed by cytometric bead array in (A) healthy controls (white) and severe asthmatics (grey) and (B) steroid‐sensitive (SS; white) and steroid‐resistant (SR; black) asthmatics pre‐ and post‐prednisolone treatment. Data assessed by Wilcoxon matched‐pairs test *P < .05, **P < .01, ***P < .001, ****P < .0001

Steroids are known to alter cytokine secretion by multiple cell types, and these cytokines in turn can regulate lymphocyte and dendritic cell populations. The concentration of a number of cytokines in the serum of healthy and severe asthmatics (pre‐ and post‐steroids) was therefore investigated to explore whether these might reflect and/or contribute to the differences observed in the frequency of Tregs and DCs in the peripheral blood. A range of cytokines was assessed; however, the cytokines IL‐1β, IL‐5, IL‐21, IFNγ and GM‐CSF were undetectable in the majority of samples assessed. Interestingly, the healthy control subjects had significantly higher IL‐13 in their serum as compared to the severe asthmatics (*P* = .0002), whilst conversely, asthma patients had higher levels of IL‐6 and IL‐8 as compared to the healthy controls (both *P* < .0001; Figure 5A). However, there was no significant difference in the quantities of cytokines in the serum of SS and SR asthmatics at baseline. Treatment of SR asthmatics with prednisolone resulted in a significant reduction in the cytokines IL‐6, IL‐8 and IL‐23p40, which was not observed in the SS; however, this may be due to the lower number of SS individuals (Figure 5B). These differences, whilst of interest, do not obviously explain the differences observed in the DC and Treg populations reported here.

## 4 DISCUSSION

The present study sought to determine whether differences could be detected in circulating leucocyte populations between disease severity‐matched SS and SR asthma patients. Our findings support our original hypothesis that SS patients demonstrate a less activated DC phenotype and that glucocorticoid treatment enhances this phenotype in SS individuals in comparison with SR patients. Specifically, we observed that a significantly higher frequency of mDCs was present in the peripheral blood of SR as compared to SS asthmatics at baseline. Although prednisolone treatment resulted in an increased frequency of circulating mDC in the SS cohort, these cells demonstrated a trend towards a less stimulatory phenotype with reduced expression of HLA‐DR and CD86, as well as a trend for an increase in the circulating Foxp3+ Treg population, which contrasted with our observations in the SR cohort. Thus, we demonstrate that significant differences resided within the mDC compartment, both at baseline and following 2 weeks of oral prednisolone therapy, which appears to reflect a more activated immune phenotype in SR as compared to SS patients. Importantly, both the SS and SR received the same regimen of oral prednisolone (14‐day course of oral prednisolone at 40 mg/1.73 m^2^/d), and there was no difference in the inhaled mean equivalent inhaled glucocorticoid dosages taken by both groups (*P* = .60) as shown previously^28^; therefore, the differences observed here are likely due to the 2‐week course of prednisolone.

Several of the outcomes examined in this study revealed no differences between SS and SR patients, or confirmed independent observations in different disease cohorts. For example, the frequencies of B cells and T cells in the peripheral blood of SS compared with SR severe asthmatics were comparable at baseline. A significant increase in the frequencies and total numbers of B cells was observed in both the SS and SR asthmatics following oral steroids which, as far as we are aware, has not been previously observed in patients with asthma. However, an earlier study observed a similar change in patients with multiple sclerosis post‐methylprednisolone treatment.^31^ Although of significant interest, as these changes occurred in both SS and SR populations, they may not be indicative of important mechanisms underlying the clinical benefit of steroids in asthma. These data are in contrast to a recent publication which showed that that there was an increase in the numbers of B and T cells in the peripheral blood 24 hours after oral steroids which reverted to baseline within 7 days,^32^ although in that study a different steroid regimen was employed as compared to our study, and a day 14 time‐point was not addressed.

A major observation arising from the current study is that a significantly higher frequency of mDCs was present in the peripheral blood of SR as compared to SS asthmatics at baseline, resulting in an increase in the ratio of mDCs/pDCs. There was also a trend for mDCs to exhibit higher expression of CD86 and HLA‐DR, but lower expression of the inhibitory receptor ILT3 than pDCs. These data suggest, along with earlier independent studies, that mDCs are better able to initiate immune responses due to their higher expression of HLA‐DR and CD86.^33^ Thus, the ratio of mDCs to pDCs may have important implications in the context of allergic and asthmatic disease. Models of allergic airway disease suggest that whilst both mDCs and pDCs take up allergen, the pDC population may preferentially induce tolerance to an inhaled allergen.^25^, ^34^ Additionally, pDCs play an important role in combating respiratory infections (a common trigger of asthma exacerbations), and in children, the number of peripheral pDCs has been reported to be inversely correlated with symptoms of lower respiratory infections,^35^ so changes in the ratio of mDC:pDC could impact asthma exacerbations.

Myeloid DCs have been shown to initiate Th2‐mediated disease leading to eosinophilic lung inflammation in mice.^23^ Recently two independent studies have also proposed that a subset of murine mDCs (CD11b+) play a vital role in the induction of Th17 responses in the mucosal tissue by secreting Th17‐polarizing molecules such as IL‐23p19, IL‐6 and TGF‐β.^36^, ^37^ These data may be of relevance to steroid refractory asthma, as IL‐17A expression is elevated in both the airways and cultured peripheral blood of more severe asthmatics, implicating IL‐17A in steroid‐resistant disease.^11^, ^13^, ^14^, ^15^, ^16^, ^17^ Asthmatics have enhanced effector responses associated with disease, and this may plausibly result from an increased frequency and/or stimulatory capacity of their mDCs; additionally, asthmatics have been demonstrated to have lower numbers of Foxp3+ Tregs.^38^, ^39^, ^40^ Our earlier study reported reduced circulating Foxp3+ T cells in SR compared to SS individuals.^18^ Here we report a trend towards an increase in the circulating Foxp3+ Treg population following oral steroid therapy in SS patients, which supports and extends earlier independent work showing increased Foxp3 expression and numbers of Foxp3+ cells following systemic steroid therapy of moderate/mild asthmatics or patients with immune thrombocytopenic purpura.^41^, ^42^ Notably this was not observed in SR asthmatics, suggesting that failure to enhance the frequency of Treg cells by steroids may be a factor contributing to clinical resistance, although further studies are warranted to strengthen this observation. These would include, in addition to studying a greater number of subjects, the inclusion of additional markers to define Tregs (such as CTLA‐4, LAP, GARP or LAG3) and assays of suppressive function. Nevertheless, these data are also consistent with the hypothesis that therapies that increase or maintain the Foxp3+ Treg population, such as vitamin D, provide greater therapeutic benefit.^18^, ^43^, ^44^

An unexpected, but interesting observation from this study is that for certain immune parameters, the response of SR asthmatics to prednisolone treatment is similar to that of SS asthmatics, such as the reduction in the number of B cells, and pDCs, whilst others clearly show a different pattern of regulation by prednisolone. This includes the effects on Tregs and on mDCs discussed further below, as well as the reduction in the serum inflammatory cytokine production in the SR but not in the SS. Thus, we conclude that steroid resistance appears to be associated with a differential response to steroids at the immunological level rather than no response.

In the present study, 2 weeks of prednisolone treatment caused a significant reduction in the frequency of total DCs, as well as pDCs, in the peripheral blood of severe asthmatics. This corresponds with other studies in patients suffering from autoimmune disease where intravenous methylprednisolone treatment caused a reduction in the frequency of plasmacytoid DCs in the peripheral blood.^25^, ^31^, ^45^ This is proposed to reflect the susceptibility of pDCs to glucocorticoid‐induced apoptosis,^46^ although additional effects of steroids on DC homing cannot be ruled out.

The most notable differential response to oral prednisolone treatment in SS and SR patients was observed at the level of mDCs. There was a significant increase in the frequency of circulating mDCs in SS asthmatics following steroid therapy alongside a trend for decreased CD86 and HLA‐DR expression on these cells, likely to render the SS mDCs less stimulatory. The reduction in the expression of CD86 and HLA‐DR is presumed to occur as a direct consequence of the prednisolone treatment on the mDCs, as has been shown previously in vitro.^47^ Notably, this change in the frequency of SS mDCs post‐steroids was not reflected in a change in the overall number, and this may therefore largely reflect the profound inhibition of pDCs post‐prednisolone. Although there was a significant fall in the median frequency and number of mDCs in the peripheral blood of SR asthmatics following steroid therapy, there was no significant effect on costimulatory molecule expression, so the cells are likely to remain highly stimulatory. We can only speculate as to the reasons for the differential effects of oral prednisolone treatment on mDC populations in SS vs SR asthma patients: it is likely to include a complex outcome of the effects of steroids on the numbers of mDCs produced in bone marrow, those recruited into lymph nodes and tissues and potentially also cell survival.^48^ Whether, and how, each of these steps is differentially regulated in SS vs SR asthma is, as far as we are aware, not clearly delineated and represents an important area for future research, beyond the scope of the present study. A limitation of the current study is that the data are from the peripheral blood only. A recent study reported differences in myeloid DC in the sputum of children with steroid‐treated asthma as compared to age‐matched healthy children, although differences pre‐ and post‐steroid treatment were not reported.^49^ To gain a full insight into what is happening in our patients, it would have been informative to have studied bronchoalveolar lavage (BAL) samples in parallel with peripheral blood from the same individual, pre‐ and post‐prednisolone treatment. However, bronchoscopy with BAL carries risks and side effects, especially in severe asthmatics, and was not therefore part of the current study design.

Another limitation of the current study is the paucity of DC markers that were utilized, and with hindsight, it would have been informative to have included, at the very least, the markers CD1c and CD141 to further differentiate mDC subpopulations into conventional type 1 (cDC1s) and conventional type 2 (cDC2s).^50^, ^51^, ^52^ Very recent studies highlight more extensive panels of antigens that can be used to further dissect mDc populations.^53^ Thus, it would have been informative to have investigated whether, and how, prednisolone targeted these different type of mDC populations.

Collectively, these data demonstrate the differential effects of 2 weeks of oral prednisolone therapy on circulating leucocyte populations as well as the contrasting effects of this therapy on SS and SR severe asthmatics with novel differences seen in mDCs between the 2 types of severe asthmatic. We believe the data from this study warrant further investigations in severe asthma as to whether the differential effects of oral prednisolone on mDCs, as well as Foxp3+ Tregs, have a translational impact as a biomarker of clinical responsiveness to steroids.

## CONFLICT OF INTEREST

The authors declare no conflict of interest.

## References

[1] Wenzel SE . Asthma phenotypes: the evolution from clinical to molecular approaches. Nat Med. 2012;18:716‐725. 22561835 10.1038/nm.2678

[2] Adcock IM , Ford PA , Bhavsar P , Ahmad T , Chung KF . Steroid resistance in asthma: mechanisms and treatment options. Curr Allergy Asthma Rep. 2008;8:171‐178. 18417060 10.1007/s11882-008-0028-4

[3] Hobbs K , Negri J , Klinnert M , Rosenwasser LJ , Borish L . Interleukin‐10 and transforming growth factor‐beta promoter polymorphisms in allergies and asthma. Am J Respir Crit Care Med. 1998;158:1958‐1962. 9847292 10.1164/ajrccm.158.6.9804011

[4] Karjalainen J , Hulkkonen J , Nieminen MM , et al. Interleukin‐10 gene promoter region polymorphism is associated with eosinophil count and circulating immunoglobulin E in adult asthma. Clin Exp Allergy. 2003;33:78‐83. 12534553 10.1046/j.1365-2222.2003.01577.x

[5] Goleva E , Searing DA , Jackson LP , Richers BN , Leung DY . Steroid requirements and immune associations with vitamin D are stronger in children than adults with asthma. J Allergy Clin Immunol. 2012;129:1243‐1251. 22330698 10.1016/j.jaci.2012.01.044 PMC3340468

[6] Li LB , Leung DY , Martin RJ , Goleva E . Inhibition of histone deacetylase 2 expression by elevated glucocorticoid receptor beta in steroid‐resistant asthma. Am J Respir Crit Care Med. 2010;182:877‐883. 20538962 10.1164/rccm.201001-0015OC PMC2970859

[7] Sutherland ER , Goleva E , Jackson LP , Stevens AD , Leung DY . Vitamin D levels, lung function, and steroid response in adult asthma. Am J Respir Crit Care Med. 2010;181:699‐704. 20075384 10.1164/rccm.200911-1710OC PMC2868500

[8] Leung DY , Martin RJ , Szefler SJ , et al. Dysregulation of interleukin 4, interleukin 5, and interferon gamma gene expression in steroid‐resistant asthma. J Exp Med. 1995;181:33‐40. 7807013 10.1084/jem.181.1.33 PMC2191836

[9] Sousa AR , Lane SJ , Cidlowski JA , Staynov DZ , Lee TH . Glucocorticoid resistance in asthma is associated with elevated in vivo expression of the glucocorticoid receptor beta‐isoform. J Allergy Clin Immunol. 2000;105:943‐950. 10808175 10.1067/mai.2000.106486

[10] Hew M , Bhavsar P , Torrego A , et al. Relative corticosteroid insensitivity of peripheral blood mononuclear cells in severe asthma. Am J Respir Crit Care Med. 2006;174:134‐141. 16614347 10.1164/rccm.200512-1930OC PMC2662905

[11] Nanzer AM , Chambers ES , Ryanna K , et al. Enhanced production of IL‐17A in patients with severe asthma is inhibited by 1alpha,25‐dihydroxyvitamin D3 in a glucocorticoid‐independent fashion. J Allergy Clin Immunol. 2013;132:297‐304 e293. 23683514 10.1016/j.jaci.2013.03.037

[12] Molet S , Hamid Q , Davoine F , et al. IL‐17 is increased in asthmatic airways and induces human bronchial fibroblasts to produce cytokines. J Allergy Clin Immunol. 2001;108:430‐438. 11544464 10.1067/mai.2001.117929

[13] Barczyk A , Pierzchala W , Sozanska E . Interleukin‐17 in sputum correlates with airway hyperresponsiveness to methacholine. Respir Med. 2003;97:726‐733. 12814161 10.1053/rmed.2003.1507

[14] Bullens DM , Truyen E , Coteur L , et al. IL‐17 mRNA in sputum of asthmatic patients: linking T cell driven inflammation and granulocytic influx? Respir Res. 2006;7:135. 17083726 10.1186/1465-9921-7-135 PMC1636037

[15] McKinley L , Alcorn JF , Peterson A , et al. TH17 cells mediate steroid‐resistant airway inflammation and airway hyperresponsiveness in mice. J Immunol. 2008;181:4089‐4097. 18768865 10.4049/jimmunol.181.6.4089 PMC3638757

[16] Al‐Ramli W , Prefontaine D , Chouiali F , et al. T(H)17‐associated cytokines (IL‐17A and IL‐17F) in severe asthma. J Allergy Clin Immunol. 2009;123:1185‐1187. 19361847 10.1016/j.jaci.2009.02.024

[17] Agache I , Ciobanu C , Agache C , Anghel M . Increased serum IL‐17 is an independent risk factor for severe asthma. Respir Med. 2010;104:1131‐1137. 20338742 10.1016/j.rmed.2010.02.018

[18] Chambers ES , Nanzer AM , Richards DF , et al. Serum 25‐dihydroxyvitamin D levels correlate with CD4(+)Foxp3(+) T‐cell numbers in moderate/severe asthma. J Allergy Clin Immunol. 2012;130:542‐544. 22656048 10.1016/j.jaci.2012.04.022

[19] Chang CC , Ciubotariu R , Manavalan JS , et al. Tolerization of dendritic cells by T(S) cells: the crucial role of inhibitory receptors ILT3 and ILT4. Nat Immunol. 2002;3:237‐243. 11875462 10.1038/ni760

[20] Penna G , Roncari A , Amuchastegui S , et al. Expression of the inhibitory receptor ILT3 on dendritic cells is dispensable for induction of CD4 + Foxp3 + regulatory T cells by 1,25‐dihydroxyvitamin D3. Blood. 2005;106:3490‐3497. 16030186 10.1182/blood-2005-05-2044

[21] Brenk M , Scheler M , Koch S , et al. Tryptophan deprivation induces inhibitory receptors ILT3 and ILT4 on dendritic cells favoring the induction of human CD4 + CD25 + Foxp3 + T regulatory cells. J Immunol. 2009;183:145‐154. 19535644 10.4049/jimmunol.0803277

[22] Holt PG , Strickland DH , Wikstrom ME , Jahnsen FL . Regulation of immunological homeostasis in the respiratory tract. Nat Rev Immunol. 2008;8:142‐152. 18204469 10.1038/nri2236

[23] Lambrecht BN , De Veerman M , Coyle AJ , Gutierrez‐Ramos JC , Thielemans K , Pauwels RA . Myeloid dendritic cells induce Th2 responses to inhaled antigen, leading to eosinophilic airway inflammation. J Clin Invest. 2000;106:551‐559. 10953030 10.1172/JCI8107 PMC380243

[24] Cella M , Facchetti F , Lanzavecchia A , Colonna M . Plasmacytoid dendritic cells activated by influenza virus and CD40L drive a potent TH1 polarization. Nat Immunol. 2000;1:305‐310. 11017101 10.1038/79747

[25] de Heer HJ , Hammad H , Soullie T , et al. Essential role of lung plasmacytoid dendritic cells in preventing asthmatic reactions to harmless inhaled antigen. J Exp Med. 2004;200:89‐98. 15238608 10.1084/jem.20040035 PMC2213319

[26] Levy ML , Thomas M , Small I , Pearce L , Pinnock H , Stephenson P . Summary of the 2008 BTS/SIGN British Guideline on the management of asthma. Primary Care Respir J. 2009;18(suppl 1):S1‐S16. 19209371 10.3132/pcrj.2008.00067 PMC6619380

[27] Corrigan CJ , Brown PH , Barnes NC , et al. Glucocorticoid resistance in chronic asthma. Glucocorticoid pharmacokinetics, glucocorticoid receptor characteristics, and inhibition of peripheral blood T cell proliferation by glucocorticoids in vitro. Am Rev Respir Dis. 1991;144:1016‐1025. 1952426 10.1164/ajrccm/144.5.1016

[28] Chambers ES , Nanzer AM , Pfeffer PE , et al. Distinct endotypes of steroid‐resistant asthma characterized by IL‐17A(high) and IFN‐gamma(high) immunophenotypes: potential benefits of calcitriol. J Allergy Clin Immunol. 2015;136:628‐637 e624. 25772594 10.1016/j.jaci.2015.01.026 PMC4559139

[29] Liu W , Putnam AL , Xu‐Yu Z , et al. CD127 expression inversely correlates with FoxP3 and suppressive function of human CD4 + T reg cells. J Exp Med. 2006;203:1701‐1711. 16818678 10.1084/jem.20060772 PMC2118339

[30] Dzionek A , Fuchs A , Schmidt P , et al. BDCA‐2, BDCA‐3, and BDCA‐4: three markers for distinct subsets of dendritic cells in human peripheral blood. J Immunol. 2000;165:6037‐6046. 11086035 10.4049/jimmunol.165.11.6037

[31] Krystyna MS , Jacek T , Sebastian R , et al. Changes in circulating dendritic cells and B‐cells in patients with multiple sclerosis relapse during corticosteroid therapy. J Neuroimmunol. 2009;207:107‐110. 19162336 10.1016/j.jneuroim.2008.11.010

[32] Olnes MJ , Kotliarov Y , Biancotto A , et al. Effects of systemically administered hydrocortisone on the human immunome. Sci Rep. 2016;6:23002. 26972611 10.1038/srep23002 PMC4789739

[33] Stock P , Akbari O , Berry G , Freeman GJ , Dekruyff RH , Umetsu DT . Induction of T helper type 1‐like regulatory cells that express Foxp3 and protect against airway hyper‐reactivity. Nat Immunol. 2004;5:1149‐1156. 15448689 10.1038/ni1122

[34] Goubier A , Dubois B , Gheit H , et al. Plasmacytoid dendritic cells mediate oral tolerance. Immunity. 2008;29:464‐475. 18789731 10.1016/j.immuni.2008.06.017 PMC3545652

[35] Upham JW , Zhang G , Rate A , et al. Plasmacytoid dendritic cells during infancy are inversely associated with childhood respiratory tract infections and wheezing. J Allergy Clin Immunol. 2009;124:707‐713 e702. 19733903 10.1016/j.jaci.2009.07.009

[36] Persson EK , Uronen‐Hansson H , Semmrich M , et al. IRF4 transcription‐factor‐dependent CD103(+)CD11b(+) dendritic cells drive mucosal T helper 17 cell differentiation. Immunity. 2013;38:958‐969. 23664832 10.1016/j.immuni.2013.03.009

[37] Schlitzer A , McGovern N , Teo P , et al. IRF4 transcription factor‐dependent CD11b+ dendritic cells in human and mouse control mucosal IL‐17 cytokine responses. Immunity. 2013;38:970‐983. 23706669 10.1016/j.immuni.2013.04.011 PMC3666057

[38] Provoost S , Maes T , van Durme YM , et al. Decreased FOXP3 protein expression in patients with asthma. Allergy. 2009;64:1539‐1546. 19392991 10.1111/j.1398-9995.2009.02056.x

[39] Vale‐Pereira S , Todo‐Bom A , Geraldes L , Schmidt‐Weber C , Akdis CA , Mota‐Pinto A . FoxP3, GATA‐3 and T‐bet expression in elderly asthma. Clin Exp Allergy. 2011;41:490‐496. 21114556 10.1111/j.1365-2222.2010.03640.x

[40] Hartl D , Koller B , Mehlhorn AT , et al. Quantitative and functional impairment of pulmonary CD4 + CD25hi regulatory T cells in pediatric asthma. J Allergy Clin Immunol. 2007;119:1258‐1266. 17412402 10.1016/j.jaci.2007.02.023

[41] Karagiannidis C , Akdis M , Holopainen P , et al. Glucocorticoids upregulate FOXP3 expression and regulatory T cells in asthma. J Allergy Clin Immunol. 2004;114:1425‐1433. 15577848 10.1016/j.jaci.2004.07.014

[42] Ling Y , Cao X , Yu Z , Ruan C . Circulating dendritic cells subsets and CD4 + Foxp3 + regulatory T cells in adult patients with chronic ITP before and after treatment with high‐dose dexamethasone. Eur J Haematol. 2007;79:310‐316. 17692100 10.1111/j.1600-0609.2007.00917.x

[43] Urry Z , Chambers ES , Xystrakis E , et al. The role of 1alpha,25‐dihydroxyvitamin D3 and cytokines in the promotion of distinct Foxp3 + and IL‐10 + CD4 + T cells. Eur J Immunol. 2012;42:2697‐2708. 22903229 10.1002/eji.201242370 PMC3471131

[44] Milliken SV , Wassall H , Lewis BJ , et al. Effects of ultraviolet light on human serum 25‐hydroxyvitamin D and systemic immune function. J Allergy Clin Immunol. 2012;129:1554‐1561. 22502796 10.1016/j.jaci.2012.03.001

[45] Navarro J , Aristimuno C , Sanchez‐Ramon S , et al. Circulating dendritic cells subsets and regulatory T‐cells at multiple sclerosis relapse: differential short‐term changes on corticosteroids therapy. J Neuroimmunol. 2006;176:153‐161. 16698092 10.1016/j.jneuroim.2006.03.022

[46] Boor PP , Metselaar HJ , Mancham S , Tilanus HW , Kusters JG , Kwekkeboom J . Prednisolone suppresses the function and promotes apoptosis of plasmacytoid dendritic cells. Am J Transplant. 2006;6:2332‐2341. 16889610 10.1111/j.1600-6143.2006.01476.x

[47] Bosma BM , Metselaar HJ , Tra WM , et al. Impairment of circulating myeloid dendritic cells in immunosuppressed liver transplant recipients. Clin Exp Immunol. 2007;149:525‐534. 17645770 10.1111/j.1365-2249.2007.03449.x PMC2219320

[48] Woltman AM , Massacrier C , de Fijter JW , Caux C , van Kooten C . Corticosteroids prevent generation of CD34 + ‐derived dermal dendritic cells but do not inhibit Langerhans cell development. J Immunol. 2002;168:6181‐6188. 12055231 10.4049/jimmunol.168.12.6181

[49] Brugha R , Mushtaq N , McCarthy NE , Stagg AJ , Grigg J . Respiratory tract dendritic cells in paediatric asthma. Clin Exp Allergy. 2015;45:624‐631. 25411998 10.1111/cea.12457

[50] Chang CC , Wright A , Punnonen J . Monocyte‐derived CD1a+ and CD1a‐ dendritic cell subsets differ in their cytokine production profiles, susceptibilities to transfection, and capacities to direct Th cell differentiation. J Immunol. 2000;165:3584‐3591. 11034359 10.4049/jimmunol.165.7.3584

[51] Piccioli D , Tavarini S , Borgogni E , et al. Functional specialization of human circulating CD16 and CD1c myeloid dendritic‐cell subsets. Blood. 2007;109:5371‐5379. 17332250 10.1182/blood-2006-08-038422

[52] Jongbloed SL , Kassianos AJ , McDonald KJ , et al. Human CD141 + (BDCA‐3)+ dendritic cells (DCs) represent a unique myeloid DC subset that cross‐presents necrotic cell antigens. J Exp Med. 2010;207:1247‐1260. 20479116 10.1084/jem.20092140 PMC2882828

[53] Guilliams M , Dutertre CA , Scott CL , et al. Unsupervised high‐dimensional analysis aligns dendritic cells across tissues and species. Immunity. 2016;45:669‐684. 27637149 10.1016/j.immuni.2016.08.015 PMC5040826

