# Supplementary material for: Dendritic cell phenotype in severe asthma reflects clinical responsiveness to glucocorticoids
Source: Clin Exp Allergy. 2017 Dec 18;48(1):13–22. doi: 10.1111/cea.13061 (PMC5767735; doi:10.1111/cea.13061)
Supplement: Supplementary file 2 [file CEA-48-13-s002.docx]

Supplementary Table 1:

| **Clinical feature (units)** |  | **Healthy Controls**  **(n=10)** | **Steroid Sensitive**  **(n=12)** | **Steroid Resistant**  **(n=23)** |
| --- | --- | --- | --- | --- |
| **Age** |  | 41 (30-61) | 49.0 (40.7-57.3) | 51.8 (45.9-57.8) |
| **Ethnic Origin** | Caucasian | 8 | 9 | 17 |
|  | African | 0 | 3 | 5 |
|  | Asian | 2 | 0 | 1 |
| **Gender** | Male/Female | 3/7 | 4/8 | 14/9 |
| **Atopic*** |  | 6 | 10 | 19 |
| **BMI (kg/m^2^)** |  |  | 31.8 (28.0-35.6) | 28.2 (26.2-30.2) |
| **Inhaled corticosteroid dosage (μg/day BDP equivalent)** |  |  | 1113 (798-1469) | 1125 (985-1520) |
| **FEV_1_ (L)** | Pre-steroid |  | 1.7 (1.4-1.9) | 1.9 (1.7-2.1) |
|  | Post-steroid |  | 2.1 (1.8-2.4) | 1.8 (1.6-2.1) |
| **FEV_1_ (% predicted)** | Pre-steroid |  | 56.0 (47.4-64.6) | 61.3 (55.3-67.3) |
|  | Post-steroid |  | 70.8 (62.6-79.0) | 59.7 (52.8-66.5) |

#### Supplementary Table 1: Patient characteristics

Where applicable, data are presented as the mean and 95% confidence interval. *Atopy was defined by skin prick testing. BDP: Beclomethasone dipropionate; BMI: body mass index. The dose of inhaled corticosteroids was calculated according to the BTS-SIGN Guideline on the management of asthma (Table 8b: Equivalent doses of inhaled steroids relative to BDP and current licensed age indications). Patients were taking beclometasone 1600 mcg/day, budenoside 1600mcg/day or fluticasone 800mcg/day.

Supplementary Figure 1:

##
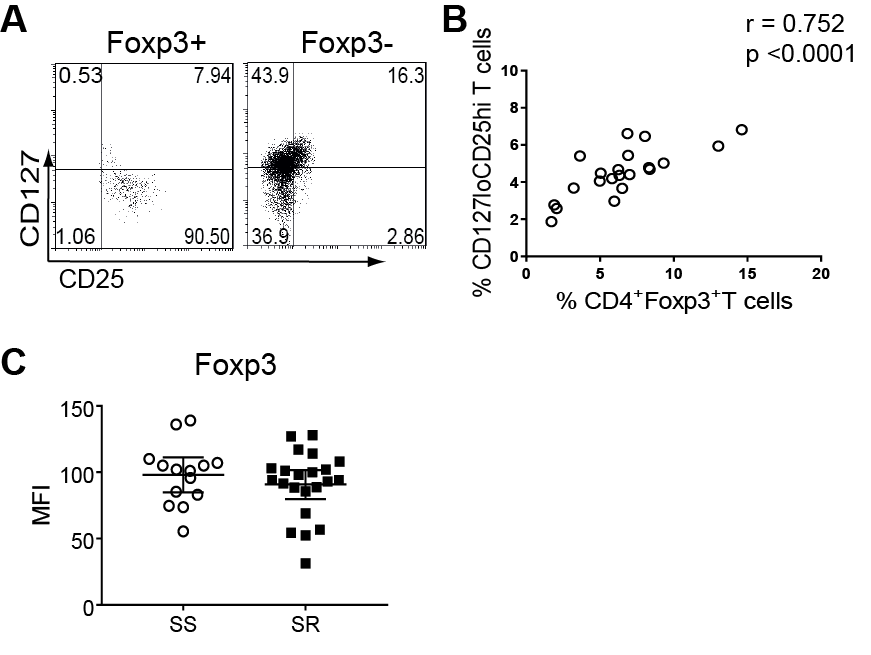


#### Supplementary Figure 1: CD4+ Foxp3+ cells correlate strongly with CD25+CD127- cells

#### A, representative staining of CD25 and CD127 in Foxp3+ (left) and Foxp3- (right) CD4+ T cells B, Correlation between the frequency of Cd4+Foxp3+ T cells and CD127loCD25hi T cells C, MFI of Foxp3 in the Foxp3+ T cells of steroids sensitive (SS; white) and resistant (SR; black) severe asthmatics. B, assessed by Pearson’s correlation test and C, assessed by Wilcoxon matched-pairs test *=p<0.05, ** p<0.01
